# Clinical Lecture on Lipoma, Cancer and Esmarch’s Tourniquet

**Published:** 1874-12

**Authors:** W. F. Westmoreland

**Affiliations:** Professor of the Principles and Clinical Surgery in Atlanta Medical College; Atlanta, Ga.


### CLINICAL LECTURE ON LIPOMA, CANCER AND ESMARCH'S TOURNIQUET.

By W. F. WESTMORELAND, M.D.,
*Professor of the Principles and Clinical Surgery in Atlanta Medical College.*

Reported by J. W. GRIGGS, M.D.

"Gentlemen: The first case that I bring before you this morning, that you have not before seen, is this good woman, who, for the past fifteen years, has been annoyed with the tumor, situated as you see between the left scapula and the spine. There has been, since she first noticed it, a gradual increase in size, until now, we find a tumor that will weigh, perhaps, two or three pounds. This growth has given her but little trouble, until quite recently, only annoying her by its interference with the movements of the scapula; and the deformity, which you see, is very perceptible through her clothing. For the past few months she suffered more or less pain in the region of the scapula and shoulder, occasionally extending itself to the entire upper extremity. The pain is not referred to the tumor; as you see, the roughest handling does not increase her suffering; but is more in the region of the scapula and shoulder. It is the result of the tumor dragging on the tissues, constantly keeping them on the stretch, and otherwise interfering with their free movements. We have here a fatty or adipose tumor, a true lipoma.

We have two varieties of fatty deposits or accumulations. One a diffusible or undefined deposit of fat, and not amounting to a disease proper. The favorite localities for this form of deposit is about the chin, nape of the neck and nates; if about the chin, making that slight deformity which you all have seen, familiarly known as double chin. This variety rarely demands assistance of the surgeon, the only inconvenience being the rather unsightly appearance. The true adipose, or fatty tumor proper, is a circumscribed deposit of fat, with a well defined capsule. Here is a fatty tumor weighing 6½ pounds, that I removed from the arm of a lady several months ago. Its immersion in alcohol has hardened its capsule, making it more dense, so that, at a distance, you can plainly see it, as it envelopes each lobule.

The surgeon more frequently meets with the fatty tumor than any other variety; and while they have their favorite localities, as the back, shoulders, etc., still there is no locality in which they may not be found. The surgeon will have but little difficulty in recognizing the fatty tumor; the only enlargements with which they are likely to be confounded are chronic or cold abscesses, aneurisms by anastomosis, and when small and in the vicinity of the scalp, may be mistaken for an atheromatous cyst. I have seen a prominent European surgeon plunge his bistoury into a fatty tumor, with the idea that he had a collection of pus. And again, I was present when a prominent American surgeon, in the presence of several hundred students, made an incision for the purpose of removing a *fatty tumor*, and was met with a bloody reception, as the supposed fatty tumor proved to be an aneurism by anastomosis. But while mistakes are sometimes made as suggested, and this by the most prominent surgeons, still we will rarely have trouble in recognizing them if we are sufficiently careful in our examinations.

Adipose tumors, as a general rule, have no attachments; one of their features being the migratory character of the tumor, from its weight. Thus a fatty deposit in the locality we find this, may be found in a few years several inches lower down. A prominent feature, and one on which I often rely, is, the possibility of readily separating them from the tissues with which they are in contact, by pushing them aside with the fingers, whatever may be their size. If, then, we have a subcutaneous tumor, that feels soft, pliant and pillowy; easily movable, of slow growth, and painless, we may feel pretty sure that we have an adipose tumor. The only treatment that promises a cure is the complete extirpation of the tumor. This is readily done in such a case as this, by a straight incision through the skin and cellular tissue. After the incision is completed, as a general rule, the knife may be laid aside, and its connections with the cellular tissue broken up with the fingers." The patient was now placed partially under the influence of ether; a straight incision, seven inches long, made through the skin and cellular tissue, and the tumor removed in a few seconds, by breaking up the connective tissue with the fingers. No vessel required the ligature, and the wound was closed with six silver sutures.

### CANCER OF THE FOOT.

"The next case that I present to you is this poor man, who for the past few months has been a great sufferer. Two years ago he noticed a small 'lump,' as he describes it, on the sole of the foot, just under the phalangeal end of the first metatarsal bone. This rapidly increased in size, until now we have, as you see, after extensive sloughing, which has occurred in the past few weeks, a tumor the size of your two fists. As you see, the mass, which consists of both bone and soft parts, is nodulated, with fissures and sinuses on every side. His sufferings for the past few months have been intense, and this, with frequent sloughs and repeated hemorrhages, has greatly exhausted him. In addition to this we find the mass infested with vermin; hundreds of maggots are constantly feeding on the diseased tissues. We have here a malignant growth, an encephaloid cancer.

We have three distinct varieties of cancer: Scirrhus or hard cancer, encephaloid or soft cancer, and epithelial cancer or epithelioma. In some of your works on surgery you will find other varieties mentioned, as colloid, melanotic, etc.; but, as I told you a few days ago, these may be properly embraced under one of the three varieties just named; that the peculiar appearances or products from which they usually derive their name, are either purely accidental, or are the result of the action of the disease upon particular tissues.

By scirrhus or hard cancer we mean that variety which hardens and condenses the tissues involved, for a time at least. A tissue attacked by scirrhus—as the mammary gland for example—becomes reduced in size, by the tissues becoming contracted and hardened, so that, when compared with the healthy gland upon the opposite side, it is perceptibly lessened in size. In encephaloid or soft cancer we have a rapid growth, the part affected rapidly increasing in size; often the tissue becomes so soft, from its great vascularity, that the surgeon is sometimes led astray by its feel, and plunges his bistoury into the part, to be rewarded by a copious flow of blood.

Epithelial cancer, or epithelioma, differs in many particulars from the other two varieties, usually commencing in the epithelial membranes, its favorite locality being the union of the mucous membrane and skin, as the lips, eyelids, nares, anus, etc. We have no time at this hour to discuss this variety of cancer as we would desire, as my time will not permit me to do more than merely mention the prominent features of each. Nor have I time to-day to discuss that more practical question, as to the origin of the disease; whether the local deposit is the result of a constitutional defect or impress, or whether it is purely a local trouble, the constitution becoming secondarily involved. The most prominent surgeons and pathologists are divided upon this subject. For myself I am satisfied that, in a number of cases at least, the disease is purely local, and that the constitutional trouble, or cancerous cachexia, is secondary. We have here an encephaloid cancer, but whether the disease commenced in the hard or soft parts, the imperfect history will not permit us to say. That both are at present involved is very evident. In addition to this degenerating mass, if we examine the patient more carefully we find the lymphatic glands, in the region of the great saphenous opening greatly enlarged. We have here not only a sympathetic enlargement, which may to some extent account for their size, but these glands are evidently infected, and partake of the same characters as the diseased foot.

What shall we do with this poor sufferer? We have here a cancer of the foot, with an enlargement of the lymphatic glands above, evidently partaking of the character of the original trouble. He is greatly debilitated by his intense suffering and repeated hemorrhages, with the cancerous cachexia well marked. Under ordinary circumstances we should not think of surgical interference, as we could only hope to give temporary relief, as I am confident the patient must sooner or later die of the disease. But in this case I have decided to remove the diseased foot by an amputation of the leg just above the ankle joint, with the perfect consciousness that in a few months, at best, should he recover from the shock of the operation, he will die from the original trouble. I operate from the fact that in his present condition he can only live a few days, and that in the most excruciating agony. I have made every effort to rid him of the maggots which infest the diseased mass, but they have burrowed so deeply into the mass and between the diseased bones, that I have not succeeded as I could desire. If he recovers from the shock of the amputation, he may have months of exemption from his intense sufferings, and will at least be rid of the horrible idea of being devoured alive.

We propose to place the patient under the influence of ether, and use this tourniquet, which has been introduced to the profession by Prof. Esmarch, to prevent the loss of the smallest quantity of blood. This tourniquet, as you see, consists of a rubber roller bandage, of sufficient length to envelop the foot and leg, and a rubber tube half an inch in diameter, with a chain attached to one end, and a hook to the other. It is long enough to encircle the limb four or five times.

The mode of applying it is as follows: Commence at the toes with the elastic roller, and apply it to the foot and leg as tight as it can be well drawn, thus driving all the blood out before the roller. When the point where we desire to compress the artery is reached, we encircle the limb with the tubing sufficiently tight to arrest the circulation in the arteries, and secure it in position by placing the hook in one of the links of the chain. After the tubing is well fastened the elastic bandage is removed, and the patient is ready for the operation. It is contended that an operation may be performed without the loss of a drachm of blood."

The tourniquet was applied, as above suggested, and the amputation of the limb made, without the loss of blood amounting to more than a drachm or two; the patient passing through the operation without perceptible shock.

